# The association between health literacy and pedestrian safety behavior among adults: a cross-sectional study

**DOI:** 10.1186/s12889-024-18441-w

**Published:** 2024-04-22

**Authors:** Hamideh Zahedi, Leila Mohammadinia, Seyedeh Leila Dehghani, Sajjad Habibzadeh, Nasrin Kheibar

**Affiliations:** 1 Student Research Committee, Department of Community Health Nursing, Nursing & Midwifery Faculty, Tabriz University of Medical Sciences, Tabriz, Iran; 2 Tabriz Health Services Management Research Center, Tabriz University of Medical Sciences, Tabriz, Iran; 3 Health in Disasters and emergencies, Department of Health Policy and Management, School of Management and Medical Informatics, Tabriz University of Medical Sciences, Tabriz, Iran; 4 Behbahan Faculty of Medical Sciences, Behbahan, Iran; 5 MSc of Medical Surgical Nursing, Tabriz University of Medical sciences, Tabriz, Iran; 6 Behbahan Faculty of Medical Sciences, Behbahan, Iran

**Keywords:** Health literacy, Pedestrian safety, Traffic incidents

## Abstract

**Introduction:**

Pedestrians are considered the most vulnerable and complex road users as human behavior constitutes one of the fundamental reasons for traffic-related incidents involving pedestrians. However, the role of health literacy as a predictor of Pedestrian safety behavior remains underexplored. Therefore, the current study was designed to examine the level of health literacy and its association with the safety behavior of adult pedestrians in the city of Tabriz.

**Methods:**

This cross-sectional analytical study was conducted among individuals aged 18 to 65 years in the metropolitan area of Tabriz from January to April 2023. Data were collected using the HELIA standard questionnaire (Health Literacy Instrument for adults), comprising 33 items across 5 domains (access, reading, understanding, appraisal, decision-making and behavior), as well as the Pedestrian Behavior Questionnaire (PBQ) consisting of 29 items. Data were analyzed using descriptive and analytical statistics (independent t-tests, ANOVA, and Pearson correlation coefficient) via SPSS-22 software.

**Results:**

Based on the results, 94% (376 individuals) had excellent health literacy levels, and their safety behavior scores were at a good level. Health literacy and safety behavior were higher among the age group of 31 to 45 years, women, married individuals, those who read books, and individuals with higher education. However, safety behavior showed no significant association with education level (*P* > 0.05). There was a significant and positive relationship between health literacy and all its domains and pedestrian safety behavior (*r* = 0.369, *P* < 0.001).

**Conclusion:**

This study underscores the significant impact of health literacy on pedestrians’ safety behavior. The findings reveal that higher levels of health literacy are associated with better safety behavior among individuals aged 18 to 63. Demographic factors such as age, gender, marital status, and education level also play a role in shaping both health literacy and safety behavior. By recognizing these relationships, interventions can be tailored to improve health literacy levels and promote safer pedestrian practices, ultimately contributing to a healthier and safer community in Tabriz city.

## Introduction

Health literacy, as defined by a set of cognitive and social abilities enabling individuals to understand and utilize health information for their betterment, plays a crucial role in empowering individuals within a person-centered care framework, and Today it has become a global goal [1–3].

The World Health Organization identifies health literacy levels as one of the major determinants of societal health and considers health literacy vital for empowerment [4]. Limited health literacy is associated with adverse health outcomes [5]. Many unfavorable outcomes in disease management and accidents result from insufficient health literacy among individuals and families [1]. Low health literacy among orthopedic injury patients resulting from accidents over a six-month period was associated with a spectrum of poor health outcomes and incomplete recovery [6]. Increasing the awareness of families with accident-involved patients plays a significant role in improvement and preventing disease recurrence [7]. Consequently, health literacy is recognized as a crucial link between various diseases and chronic or acute health conditions such as traffic injuries [8].

One of these significant medical incidents is traffic accidents. According to the World Health Organization, annually, 1.3 million people die due to traffic accidents, and 20 to 50 million suffer severe injuries requiring prolonged and expensive medical care. The WHO predicts that by 2030, traffic accidents will become the fifth leading cause of death globally, highlighting the increasing importance of preventing these incidents [9, 10]. In the European Union countries, in 2018, 40% of fatalities in road traffic accidents involved pedestrians out of 9,500 casualties in urban road accidents [11]. In Iran, over 30% of road accidents involve pedestrians [12], considering pedestrians as vulnerable road users [12] exposed to severe health consequences in the event of traffic accidents [13]. Furthermore, among road users, pedestrians are the most adaptable and can exhibit the quickest reactions. However, their behavior is unpredictable and cannot be effectively regulated by rules [14]. Given that accidents are often unpredictable, the risk of road accidents always exists [15]. Therefore, enhancing safety and safe behavior, including improving the awareness of road users such as pedestrians, is crucial.

Several factors influence pedestrian-related traffic accidents, with human behavior being the most relevant [16]. Studies have indicated that one-third of pedestrians exhibit unsafe behaviors and distractions, with the most common causes being the use of headphones, texting, and talking on mobile phones [12]. While health literacy is well recognized as “the degree to which individuals have the capacity to obtain, process, and understand basic health information and services needed to make appropriate health decisions,” it is rarely studied in the context of accidents, especially traffic accidents [6].

In Iran, the establishment of a capable and healthy society in the 20-year vision plan of the Islamic Republic of Iran until 2025 has been envisaged and announced. Assessing health literacy provides a means to evaluate individuals’ understanding and insights into their health management capabilities [14]. While health literacy enhances individuals’ abilities to adopt necessary care and understand and interpret health information for a healthy lifestyle, its role as a predictor of pedestrian safety behavior remains understudied. Therefore, the current study aimed to investigate the level of health literacy and its association with pedestrian safety behavior among a sample of pedestrians aged 18 to 65 in Tabriz city.

## Methods

This cross-sectional analytical study was conducted among adults in the metropolitan area of Tabriz from January to April 2023. Individuals aged between 18 and 65 years, possessing literacy in reading and writing, and engaging in daily walking were included in the study.

The statistical population of Tabriz city for this age group was approximately 1,345,044 individuals based on the census conducted in the year 1396 in the Persian calendar (approximately corresponds to the years 2017–2018 in the Gregorian calendar). Considering the sample size estimation using the Morgan table, a minimum sample size of 384 individuals was estimated. Additionally, according to the study by Tavousi et al. and colleagues and based on the formula for determining sample size, as further explained, a sample size of 379 individuals was estimated [17].

*p* = 0.56 q = 0.44

Z = 1. 96

d = 0.05

n = Z^2^ (pq) ÷ d^2^

*n* = 379

### Data collection tools

#### The data were collected using three questionnaires

A. **Demographic Variables Questionnaire**: This questionnaire gathered demographic information, including gender, age, marital status, education level, access to health-related content, the frequency of walking days per week, hours of walking per day, and mode of transportation.
B. **Iranian Health Literacy Assessment Questionnaire (HELIA)**: Health Literacy Instrument for adults): This questionnaire, designed by Montazeri and colleagues (1393), was utilized to evaluate health literacy levels. It comprises 33 items divided into five domains: access (6 items), reading (4 items), comprehension (7 items), appraisal (4 items), decision-making and behavior (12 items). Responses were rated on a five-point Likert scale ranging from 1 to 5. The total score ranged from 33 to 165, indicating inadequate health literacy (0–50), marginal health literacy (51–66), adequate health literacy (67–84), and excellent health literacy (85–100). The content validity was confirmed in the study by Montazeri et al., and the questionnaire’s reliability was obtained using Cronbach’s alpha of 0.89 [17].

##### Pedestrian Safety Behavior Questionnaire (PBQ)

The evaluation of pedestrian safety behavior was conducted using the questionnaire designed by Sadeghi et al. (2018) for the Iranian population. This questionnaire comprised 29 items. Content validity was confirmed in this study, and the questionnaire’s reliability was established with a Cronbach’s alpha of 0.84. Responses were collected using a five-point Likert scale (never, rarely, sometimes, often, always) [18].

### Data collection procedure

The data were collected using a convenience sampling method with the mentioned questionnaires through online methods. Online questionnaires were designed using the website platform https://avalform.com/, and their links were shared through various virtual networks (email, WhatsApp, Eitaa and Telegram) accessible to the target group.

As some middle-aged individuals might not use or have access to virtual networks, two community health nurses (H.Z & N.KH) conducted face-to-face interviews to explain the study’s objectives, the significance of the subject matter, and the importance of confidentiality. After the explanation, the questionnaire was completed online.

Follow-ups were conducted weekly by sending reminder messages through relevant channels to encourage completion of the questionnaires. The sampling continued for up to six months until the desired sample size was achieved. The collected data were transferred to data analysis software (SPSS-22) for analysis.

Considering that the normality of the data was confirmed using the Kolmogorov-Smirnov test, parametric tests were used to compare the means in the groups. Statistical analyses included descriptive statistics (mean, standard deviation, and percentage) and analytical statistics (independent t-test, ANOVA (Tukey Post Hoc), and Pearson correlation coefficient).

This study received approval from the Tabriz Health Services Management Research Center, Tabriz University of Medical Sciences, Tabriz, Iran (Approval Code: IR.TBZMED.REC.1401.591). Prior to participation, informed consent was obtained from all participants, and they were assured of the confidentiality and anonymity of their data.

## Results

In this study, 400 individuals were included through convenience sampling using the online platform https://avalform.com. The statistical population consisted of individuals aged between 18 and 63 years residing in Tabriz city. The mean total score of health literacy was 130.9 ± 26.1 out of 165. Among the participants, 94% (376 individuals) demonstrated excellent health literacy, while 5.4% (18 individuals) exhibited a good level of health literacy. The results revealed variations in health literacy among individuals based on their demographic characteristics.

According to the ANOVA statistical test, there was a significant and meaningful difference in health literacy across different age groups (*P* < 0.001). Post hoc tests showed this difference was mainly due to variations in health literacy scores between the age group of 31–45 years and those above 60 years when compared to other age groups. The highest health literacy score was observed in the age group of 31–45 years (140.3 ± 23.9). Based on the independent t-test, there was a statistically significant difference in health literacy between women and men (*P* = 0.049), with women demonstrating significantly higher health literacy scores than men (132.9 ± 24.1). Additionally, according to the independent t-test between single and married individuals, a statistically significant difference in terms of health literacy existed (*P* < 0.001), with married individuals exhibiting higher health literacy scores than single individuals (135.5 ± 23.9).

Regarding the ANOVA statistical test, there was a statistically significant difference in health literacy across different education levels (*P* < 0.001). Post hoc tests indicated that this difference was due to variations in health literacy scores between individuals with university education and others. The highest health literacy score was observed in individuals with postgraduate degrees (153.9 ± 12.5), and this increased with higher levels of educational attainment (see Table 1).

**Table 1:** Mean score of health literacy according to demographic variables

Variable		N (%)	health literacy score (Mean ± SD^a^)	^*^P-value
**Age**	18–30	184(46)	126. 4 ± 22. 8	< 0. 001^b^
	31–45	135(33. 8)	140. 3 ± 23. 9	
	46–60	73(18. 3)	126. 9 ± 30. 1	
	> 60	8 (2)	117. 1 ± 47. 1	
**Total**	-	400(100%)	130. 9 ± 26. 1	
**Sex**	Male	150(37. 5)	127. 6 ± 28. 9	0. 049^c^
	Female	250(62. 5)	132. 9 ± 24. 1	
**Total**	-	400(100%)	130. 9 ± 26. 1	
**Marital status**	Married	194(48. 5)	135. 5 ± 23. 9	< 0. 001^c^
	Single	206(51. 5)	125. 6 ± 26. 9	
**Total**	-	400(100%)	130. 9 ± 26. 1	
**Education**	under diploma	66(16. 5)	98. 8 ± 23. 7	< 0. 001^b^
	Diploma	97(24. 3)	126. 6 ± 17. 3	
	upper diploma and bachelor’s degree	146(36. 5)	136. 4 ± 21. 9	

The highest health literacy was associated with individuals who engaged in walking for more than 120 min per day and 4–7 days per week. In terms of the source of information, individuals who utilized books tended to have higher health literacy scores (see Table 2).

**Table 2:** Mean score of health literacy according to Physical activity and information acquisition

Variable		N (%)	health literacy score (Mean ± SD^a^)	^*^P-value
**Duration of walking per day(minute)**	< 30	115(28. 7)	124. 8 ± 26. 2	0. 002^b^
	30–60	115(28. 7)	129. 5 ± 26. 8	
	60–120	121(30. 3)	134. 9 ± 26. 2	
	> 120	49(12. 3)	137. 9 ± 16. 3	
**Total**	-	400(100%)	130. 9 ± 26. 1	
**Walking a week(day)**	0–1	116(28. 9)	132. 7 ± 26. 5	< 0. 001^b^
	2–3	153(38. 3)	136. 1 ± 23. 1	
	4–7	131(32. 8)	137. 1 ± 25. 1	
**Total**	-	400(100%)	130. 9 ± 26. 1	
**A source of health information**	Internet	250(62. 5)	139. 4 ± 19. 4	< 0. 001^b^
	Personal	108 (27)	116. 1 ± 31. 4	
	Friends	12 (3)	121. 8 ± 21. 9	
	Radio	22(5. 5)	107. 8 ± 21. 4	
	Book	8 (2)	140. 8 ± 18. 7	
**Total**	-	400(100%)	130. 9 ± 26. 1	
**transportation vehicle**	private car	160 (40)	131. 9 ± 26. 1	0. 06^b^
	Taxi	120 (30)	130. 9 ± 25. 1	
	Big vehicle	90(22.5)	130. 9 ± 24. 1	
	Motorcycle	15(3.75)	128. 9 ± 21. 1	
	Walking	15(3.75)	132. 9 ± 22. 1	
**Total**	-	400(100%)	130. 9 ± 26. 1	

^a^ Standard Deviation

^b^ One Way Anova

^c^ Independent sample t-test

^*^The significance level is 0.05

The score for pedestrians’ safety behavior ranged from 80 to 117, with an average safety behavior score of 101.7 out of 145.

The results indicated that the score for pedestrians’ safety behavior varied based on demographic characteristics. According to the ANOVA statistical test, there was a statistically significant difference in safety behavior scores among different age groups (*P* = 0.001). Post hoc tests indicated that this difference was due to variations in safety behavior scores between the age group of 45 − 31 and the age group of 30 − 18 years. The highest safety behavior score was associated with the age group of 45 − 31 years (103.8 ± 7.7).

Based on independent t-tests, there was a statistically significant difference in safety behavior between men and women (*P* = 0.002), with women demonstrating significantly higher safety behavior scores than men (102.7 ± 8.1). Additionally, between single and married individuals, there was a statistically significant difference in safety behavior scores (*P* = 0.03), with married individuals scoring higher in safety behavior than single individuals (102.6 ± 7.7).

Regarding the ANOVA statistical test, there was no statistically significant difference in safety behavior scores across different education levels (*P* = 0.3). The highest safety behavior score was observed in individuals with a high school diploma (103.1 ± 9.2) (see Table 3).

**Table 3:** Mean score of Pedestrian Safety Behavior according to demographic variables

Variable		N (%)	(Mean ± SD^a^)^b^	*P-value
**Age**	18–30	184(46)	100.6 ± 8.2	0.001^c^
	31–45	135(33. 8)	103.8 ± 7.7	
	46–60	73(18. 3)	101.2 ± 7.9	
	> 60	8 (2)	97.3 ± 2.5	
**Total**	-	400(100%)	130.9 ± 26.1	
**Sex**	Male	150(37. 5)	100.1 ± 7.7	0.002^d^
	Female	250(62. 5)	102.7 ± 8.1	
**Total**	-	400(100%)	130.9 ± 26.1	
**Marital status**	Married	194(48. 5)	102.6 ± 7.7	0.03^d^
	Single	206(51. 5)	100.9 ± 8.3	
**Total**	-	400(100%)	130.9 ± 26.1	
**Education**	under diploma	66(16. 5)	99.8 ± 7.5	0.30^c^
	Diploma	97(24. 3)	103.1 ± 9.2	
	upper diploma and bachelor’s degree	146(36. 5)	101.1 ± 7.6	
	Masters and PhD	91(22. 8)	102.8 ± 7.5	
**Total**	**-**	400(100%)	130.9 ± 26.1	

^a^ Standard Deviation

^b^ Pedestrian Safety Behavior score

^c^ One Way Anova

^d^ Independent sample t-test

^*^The significance level is 0.05

Based on the results of this study, there was a significant direct relationship between health literacy and pedestrians’ safety behavior, which was statistically significant (*r* = 0.369, *P* < 0.001). This signifies that an increase in health literacy significantly enhances pedestrians’ safety behavior.

Furthermore, there was a statistically significant direct relationship between all aspects of health literacy and pedestrians’ safety behavior (*P* < 0.5) (see Table 4).

**Table 4:** The relationship of Pedestrian Safety Behavior with health literacy and items among adults in Tabriz city in 2023

Variable		r	*P-value
**Pedestrian Safety Behavior**	**health literacy score**	0. 369	< 0. 001
	**Reading**	0. 221	< 0. 001
	**Access**	0. 145	0. 004
	**Understanding**	0. 266	< 0. 001
	**Appraisal**	0. 263	< 0. 001
	**decision-making and behavior**	0. 473	< 0. 001

**r** Pearson Correlation Coefficient

^*^The significance level is 0.05

Based on the results presented in Table 5, the average score of health literacy and its components, as well as the average score of pedestrians’ safety behavior, were at a good level (see Table 5).

**Table 5:** The mean score of health literacy and its items and Pedestrian Safety Behavior

Variable	health literacy score (from 165)	Reading score (from 20)	access score (from 30)	Understanding score (from 35)	Appraisal score (from 20)	decision-making and behavior score (from 60)	Pedestrian safety behavior score (from 145)
**Mean**	130. 9	15. 4	23. 7	27. 9	15. 5	48. 4	101. 7
^**a**^ **SD**	26. 1	3. 6	5. 4	6. 7	3. 9	9. 9	8. 1

^a^ Standard Deviation

## Discussion

The present study aimed to investigate the level of health literacy and its association with pedestrian safety among adults in the metropolitan city of Tabriz. Regarding demographic characteristics, the study results indicated that health literacy is associated with some demographic characteristics. The first identified factor is age, where the highest health literacy score was related to the age group of 31–45 years. In this regard, in a previous study, it was found that with increasing age, individuals had lower health literacy scores [19–21]. The second identified factor is gender, indicating that women have higher health literacy than men. The current study’s findings align with previous studies that showed men had a higher chance of having insufficient health literacy [20, 22]. However, a study conducted in Ghana demonstrated that men had higher health literacy than women [23]. Gender differences in health literacy can be influenced by various factors. While it is crucial to consider individual experiences, some potential reasons include social norms and expectations, cultural influences, access to healthcare and education, and communication styles.

The third identified factor is marital status, where married individuals obtained higher health literacy scores compared to unmarried individuals. The results of the study by Papi et al. are consistent with the current study’s findings [24]. Another study showed that married and single individuals achieved higher health literacy scores than divorced or widowed individuals, as the latter lacked motivation for health education classes due to mental issues [25]. The higher level of health literacy in married individuals might be explained by the fact that in a committed relationship, having mutual responsibilities, including health care decisions and information seeking, and the support of partners likely influence the comprehension and guidance of health information.

The fourth factor is individuals’ educational level, where those with university education had higher levels of health literacy. Previous studies confirm the findings of the present study [22, 26]. Lastly, the hours of walking per day and per week were significant, indicating that individuals who walked more than 120 min per day and 3–7 days per week demonstrated higher health literacy. According to the investigations, no studies were found that measured the relationship between health literacy and the amount of walking. To confirm the findings of the present study, it is suggested to conduct further research in this direction.

“Furthermore, concerning the demographic characteristics and the pedestrian safety behaviors, the present study results indicated that pedestrian safety behavior is associated with some demographic characteristics. The first identified factor is individuals’ age. Accordingly, individuals in the age group of 31–45 demonstrated higher pedestrian safety behavior scores compared to those aged 18–30. The current study’s findings align with previous studies showing that pedestrians aged 35–45 exhibited positive safety behaviors [27], while younger pedestrians tended to commit more violations and errors [28]. The second identified factor is gender, wherein female pedestrians showed higher scores in pedestrian safety behavior than males. The present study’s results are consistent with previous studies conducted in Iran, indicating that women significantly outperformed men in three pedestrian traffic behavior domains (i.e., non-violation, non-distraction, and non-aggressive behavior) [29]. Additionally, another study revealed that the violation rate among male pedestrians is 1.47 times higher than among females [30]. This might be explained by women being more cautious in driving and paying closer attention. However, a study in China reported contradictory results, indicating that women are more prone to deviation from traffic rules and guidelines [31]. Another study demonstrated that gender had no influence on safety behaviors among pedestrians [32].

The third identified factor is marital status, where married individuals exhibited higher pedestrian safety behavior scores compared to singles. Previous studies align with the current study’s findings, highlighting that being married is a determining factor for safe traffic behavior [29, 33]. Therefore, implementing educational interventions through various media and environmental interventions by different organizations seems essential to improving safe behavior among young and single pedestrians, especially among men.

Based on the current study’s results, the majority of individuals had an excellent level of health literacy. Moreover, in assessing the dimensions of health literacy, almost all health literacy dimensions obtained similar scores, with the highest mean health literacy score related to decision-making and the lowest related to reading and evaluation. The present study’s findings are in line with a study by Joveini et al. conducted in Sabzevar, Iran, showing that 84% of individuals had an excellent overall health literacy score. Similarly, in that study, understanding received the highest mean score, while reading received the lowest score among health literacy dimensions [25]. However, the current study contradicts the results of Eftekhari et al., which were conducted on individuals aged 18–65 visiting comprehensive health centers in Neyshabur, Iran, where only 5.6% of participants had an excellent level of health literacy, with most participants having insufficient health literacy. In the study of Eftekhari et al., the highest frequency of desirable health literacy was related to the dimension of understanding and the lowest frequency was related to the dimension of access [34]. In another study conducted in Denmark among the general population, despite the relatively educated population, the prevalence of insufficient health literacy is high, with only 9.60% of people having sufficient health literacy and the rest having insufficient or problematic health literacy [22]. In a study conducted in Turkey, 85.1% of people had insufficient health literacy [35]. On the other hand, in a systematic review of studies conducted in England and Germany, out of 8 studies reviewed, only one reported adequate health literacy, and five studies reported limited health literacy [36].

Regarding pedestrian safety behavior, the present study revealed a good level of pedestrian safety behavior, with an average safety behavior score of 101.7 out of 145. Concerns about pedestrian fatalities worldwide, especially in low-income countries like Iran, are increasing. Notably, pedestrian deaths account for over 22% of total accident fatalities in Iran, which is higher in metropolitan areas [37]. In this regard, Zaareh et al. conducted a study aiming to examine unsafe behaviors among adult pedestrians in Iran. The most prevalent unsafe behaviors included failure to look left and right while crossing streets, failure to use pedestrian bridges, failure to use pedestrian crossings, and crossing between vehicles. Overall, the results indicated that pedestrians in cities might engage in risky behaviors jeopardizing their lives [38]. These behaviors need to be scrutinized for the targeted design and implementation of intervention programs to reduce pedestrian injuries and fatalities. Another study conducted in Iran among people aged over 60 showed that over 90% of pedestrians exhibited unsafe traffic behaviors, including traffic violations, non-compliance with traffic rules, aggressive behavior, and feeling anxious while crossing streets [33]. Results from a study in Birmingham showed that one-third of pedestrians exhibit unsafe behaviors and distractions, with the most common causes being the use of headphones, texting, and talking on mobile phones [12]. In general, pedestrian violations play a significant role in pedestrian-vehicle collisions’ frequency and severity. Accordingly, an analysis revealed that factors such as waiting time next to the street, traffic volume, walking speed, pedestrian distractions, bus stops, schools, and street parking are key factors increasing the likelihood of pedestrian violations [39].

Regarding the relationship between health literacy and pedestrian safety, the results indicate a significant and positive correlation between health literacy and pedestrian safety behavior. In this regard, a study by Pawloski et al. examining road traffic injuries in Poland highlighted non-compliance and lack of attention to traffic regulations as major causes of these statistics, emphasizing the necessity of cultural and behavioral changes in society and an increase in health literacy levels [9]. Study on the differences in knowledge and counseling among clinical groups concerning pedestrian safety and adolescent driving was highly variable. The utilization of software programs for enhancing knowledge and injury prevention behaviors among families regarding road traffic and pedestrian safety was effective [40]. Another study by Miszkowicz et al. among mothers with school-aged children aimed at health literacy as a predictor of road traffic injury prevention. The findings revealed a weak positive correlation between maternal knowledge and road traffic injury prevention. The study indicated that the level of health literacy influences mothers’ knowledge of preventing road traffic injuries [41]. Moreover, in disease management and accidents, many unfavorable outcomes stem from insufficient health literacy among people and families. Individuals with adequate health literacy can make appropriate decisions regarding their health [2].

## Conclusion

Pedestrians with higher education levels show better safety behavior. Health literacy is linked to safety behavior, highlighting the importance of health education for promoting pedestrian safety. Demographic factors like age, gender, marital status, and education level should be considered when addressing health literacy and safety behavior. Targeting specific groups with lower health literacy or safety behavior scores can improve overall pedestrian safety. Enhancing health literacy can lead to positive outcomes in promoting safe pedestrian practices. Further research and interventions based on these findings could create a safer environment for pedestrians in Tabriz city.

### Limitations

Based on the assessments conducted the present study is the first to examine the relationship between health literacy and pedestrian safety. However, like other studies, this research has limitations. As a cross-sectional study, it has a weak ability to establish definitive cause-and-effect relationships. Therefore, to confirm the findings of this study, it is suggested that future studies using various research methods, including intervention studies or trials, and even descriptive designs with larger sample sizes in several provinces should be conducted. Secondly, the data were collected using self-reported questionnaires. In all self-reported questionnaires, social desirability can influence the accuracy of the provided responses. To mitigate this bias, participants were assured of the anonymity of the survey. Finally, our study was conducted among the general population aged over 18 in the metropolitan city of Tabriz. This is significant because different patterns and results might be reported in the adolescent and young adult groups. Also, when generalizing the results to other cities in Iran and abroad, cultural and climatic similarities should be taken into account. Conducting further research and studies specifically focused on this topic will be beneficial for a more comprehensive understanding of the relationship between health literacy and pedestrian safety.

## Data Availability

For ethical reasons, the data sets generated and analyzed as part of the current study are not publicly accessible but can be requested from the corresponding author upon justified request.
